# Risk of Adverse Pregnancy Outcomes in Young Women with Thyroid Cancer: A Systematic Review and Meta-Analysis

**DOI:** 10.3390/cancers14102382

**Published:** 2022-05-12

**Authors:** Shinje Moon, Ka Hee Yi, Young Joo Park

**Affiliations:** 1 Department of Internal Medicine, Hallym University College of Medicine, Seoul 07440, Korea; sinjei1129@gmail.com; 2 Department of Internal Medicine, Seoul Metropolitan Government-Seoul National University Boramae Medical Center, Seoul 07061, Korea; khyi@snu.ac.kr; 3 Department of Internal Medicine, Seoul National University College of Medicine, Seoul 03080, Korea; 4 Department of Molecular Medicine and Biopharmaceutical Sciences, Graduate School of Convergence Science and Technology, Seoul National University, Seoul 03080, Korea; 5 Genomic Medicine Institute, Medical Research Center, Seoul National University College of Medicine, Seoul 03080, Korea

**Keywords:** thyroid cancer, radioactive iodine treatment, pregnancy outcomes, adverse effects

## Abstract

**Simple Summary:**

This meta-analysis of 22 articles investigated whether thyroidectomy or radioactive iodine treatment (RAIT) in patients with differentiated thyroid cancer was associated with an increase in adverse pregnancy outcomes, such as miscarriage, preterm delivery, and congenital malformations. The results of this meta-analysis suggest that thyroid cancer treatment, including RAIT, is not associated with an increased risk of adverse pregnancy outcomes, including miscarriage, preterm labor, and congenital anomalies.

**Abstract:**

This meta-analysis investigated whether thyroidectomy or radioactive iodine treatment (RAIT) in patients with differentiated thyroid cancer (DTC) was associated with an increase in adverse pregnancy outcomes, such as miscarriage, preterm delivery, and congenital malformations. A total of 22 articles (5 case-control and 17 case series studies) from 1262 studies identified through a literature search in the PubMed and EMBASE databases from inception up to 13 September 2021 were included. In patients with DTC who underwent thyroidectomy, the event rates for miscarriage, preterm labor, and congenital anomalies were 0.07 (95% confidence interval [CI], 0.05–0.11; 17 studies), 0.07 (95% CI, 0.05–0.09; 14 studies), and 0.03 (95% CI, 0.02–0.06; 17 studies), respectively. These results are similar to those previously reported in the general population. The risk of miscarriage or abortion was increased in patients with DTC when compared with controls without DTC (odds ratio [OR], 1.80; 95% CI, 1.28–2.53; I^2^ = 33%; 3 studies), while the OR values for preterm labor and the presence of congenital anomalies were 1.22 (95% CI, 0.90–1.66; I^2^ = 62%; five studies) and 0.73 (95% CI, 0.39–1.38; I^2^ = 0%; two studies) respectively, which showed no statistical significance. A subgroup analysis of patients with DTC according to RAIT revealed that the risk of miscarriage, preterm labor, or congenital anomalies was not increased in the RAIT group when compared with patients without RAIT. The results of this meta-analysis suggest that thyroid cancer treatment, including RAIT, is not associated with an increased risk of adverse pregnancy outcomes, including miscarriage, preterm labor, and congenital anomalies.

## 1. Introduction

According to recent cancer statistics, approximately 75% of differentiated thyroid cancer (DTC) occurs in women, with the highest incidence found in those aged 50–59 years in the United States [1]. DTC is one of the most common cancers affecting women aged 15–39 years, and recent studies have shown an increase in the incidence of DTC in this population [2]. Most DTCs have a good prognosis with a 5-year survival rate of >98% [3,4]. Despite the low mortality, recurrence is relatively common [5].

A Korean nationwide study involving patients with DTC smaller than 2 cm reported overall 5- and 10-year recurrence rates of 4.5% and 9.2%, respectively [6]. A recent meta-analysis of 31 studies on low-risk DTC showed that the pooled 10-year recurrence rate was 9.0% in patients who underwent hemithyroidectomy and 7.4% in those who underwent total thyroidectomy [7]. Moreover, recurrence more frequently occurs in younger patients, although their survival is rarely affected [8,9]. Therefore, concerns regarding undesirable health outcomes related to DTC treatment modalities, particularly in younger patients, are increasing [10].

Thyroidectomy and radioactive iodine treatment (RAIT) are standard treatments for DTC [5]. All patients who undergo total thyroidectomy and more than half of patients who undergo less-than-total thyroidectomy receive thyroid hormone replacement therapy after thyroidectomy [5], and some of them require thyroid hormone suppression therapy. Thus, the adverse effects of thyroid hormone over- or under-replacement after thyroidectomy can last a lifetime [5]. The importance of adequate thyroid hormonal status, particularly in pregnant women, has been emphasized in association with pregnancy outcomes [11]. 

RAIT has been administered in 45–55% of all patients with DTC [12,13,14], and several adverse events have been reported [15,16], including temporary amenorrhea/oligomenorrhea, earlier onset of menopause, infertility, and adverse pregnancy outcomes [16,17,18]. A recent meta-analysis involving four studies reported that RAIT was not associated with a decreased birthrate; however, there was significant heterogeneity among the included studies [18,19]. Considering the progressively increasing incidence of DTC in younger women, the association of thyroid cancer treatment with adverse pregnancy outcomes and infertility is one of the important issues for women with DTC. 

Several case-control [14,20] and case series [21,22] studies have attempted to investigate such associations; nonetheless, the evidence regarding adverse pregnancy outcomes in patients with DTC is still inconclusive [20]. Therefore, we aimed to evaluate the incidence of adverse pregnancy outcomes, including miscarriage, preterm delivery, and congenital malformations, in patients with thyroid cancer and to investigate whether patients with DTC had an increased risk of adverse pregnancy outcomes compared with those without DTC by conducting a meticulous meta-analysis and systematic review.

## 2. Materials and Methods

### 2.1. Search Strategy

The present study was registered in the “International Platform of Registered Systematic Review and Meta-Analysis Protocols” in 2022 (INPLASY202240075) and was conducted according to PRISMA guidelines.

A literature search was conducted according to the protocol recommended by the Preferred Reporting Items for Systematic Reviews and Meta-Analyses (Table S1). Two investigators (S.M. and Y.J.P.) refined data extraction tables prior to data extraction. These two investigators searched citation databases, including PubMed and EMBASE (from inception until 13 September 2021), and extracted data independently using the predefined tables for data extraction. Discrepancies were resolved by discussion with a third investigator (K.H.Y.). Search terms included combinations of the following: (“Pregnancy”), (“Infertility”), (“Birth”), (“abortion”), (“miscarriage”), (“preterm”) OR (“ovary”) AND (“thyroid cancer”) in the title or abstract.

### 2.2. Study Selection

Studies with the following characteristics were included: (1) population: pregnant women aged ≥20 years; (2) intervention: total thyroidectomy with/without RAIT or subtotal thyroidectomy; (3) comparators: pregnant women without thyroid cancer in case-control studies (there were no comparators in case series studies); (4) outcomes: miscarriage or abortion, preterm delivery, and congenital malformations; and (5) study design: case-control or case series designs using a registry of patients with thyroid cancer. 

We excluded studies with the following characteristics: (1) articles on animal studies or in vivo experiments; (2) articles that included only abstracts; (3) non-original articles, including expert opinions or reviews; and (4) studies with insufficient information on adverse pregnancy outcomes.

### 2.3. Quality Assessment

The Newcastle–Ottawa Quality Assessment Scale was used to assess the methodological quality of case-control studies [23]. Based on eight items, a maximum of nine points were awarded to each study, categorized into three broad perspectives: selection, comparability, and exposure. Studies with a score of 7 or higher were defined as having a low risk of bias [24]; case series study designs were considered to have a high risk of bias owing to the lack of control data. Any discrepancies were resolved through a discussion with a third investigator (K.H.Y.).

### 2.4. Data Analyses and Statistical Methods

The event rate of studies was estimated based on the incidence of adverse pregnancy outcomes in patients with thyroid cancer. The pooled event rate was calculated for each adverse pregnancy outcome using a random-effects model. Odds ratios (ORs) and 95% confidence interval (CIs) were computed for each study using the Mantel–Haenszel method. Pooled ORs were calculated for each adverse pregnancy outcome based on thyroid cancer treatment using a random-effects model.

The heterogeneity among the studies was tested using Higgins’ I^2^ statistic, where I^2^ ≥ 50% indicated heterogeneity. Publication bias was tested using Egger’s test and a funnel plot. In addition, to evaluate the effect of RAIT on adverse pregnancy outcomes, we conducted a subgroup analysis of studies that included patients who received RAIT. All statistical analyses and graphical presentations were conducted using the Comprehensive Meta-Analysis software version 3 (Biostat Inc., Englewood, NJ, USA).

## 3. Results

### 3.1. Study Characteristics

**Figure 1:**
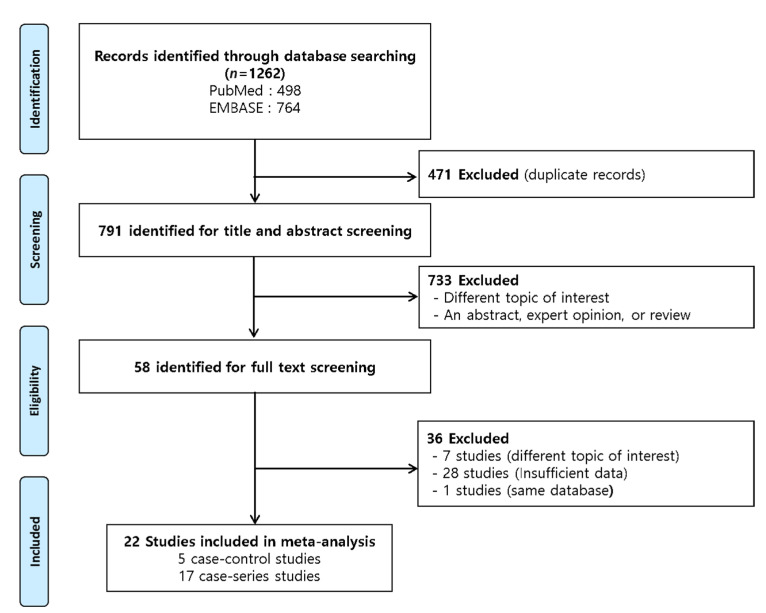
Scheme of the search strategy.

The literature search yielded 1262 studies (PubMed: 498, EMBASE: 764). Following the exclusion of 471 duplicate studies and 769 studies that did not meet the inclusion criteria or had insufficient data, a total of 22 studies [14,20,21,22,25,26,27,28,29,30,31,32,33,34,35,36,37,38,39,40,41,42] were finally included in the meta-analysis (Figure 1). The characteristics of each study are summarized in Table 1.

### 3.2. Risk of Bias Assessment

The Newcastle–Ottawa Quality Assessment Scale for case-control studies revealed that four [25,27,30,42] out of five case-control studies had a low or moderate risk. (Table S2). One study [31] had a high risk of bias. Five studies classified as case series studies [14,20,29,35,36] were included in subgroup analysis, which compared the risk of adverse pregnant outcomes in patients with DTC according to RAIT. Two studies [14,20] had a low or moderate risk, and three studies [29,35,36] had a high risk of bias.

### 3.3. Thyroid Cancer Treatment and Risk of Miscarriage or Abortion

**Figure 2:**
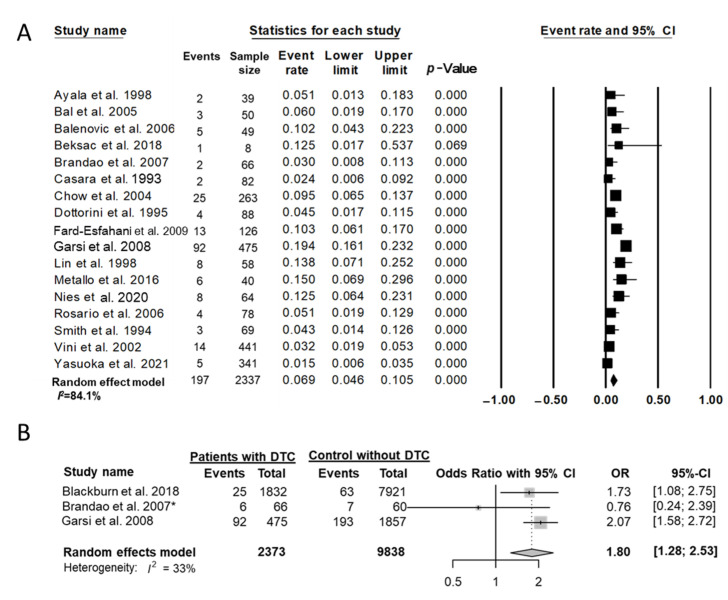
Effect of treatment of differentiated thyroid cancer on miscarriage. (**A**) Event rate among cases of pregnancy and (**B**) the OR between patients with DTC and controls without differentiated thyroid cancer; * abortion. Studies referenced in the figure are: [21,22,25,26,28,29,30,31,32,33,34,35,36,37,39,40,41,42].

Nine studies [22,25,29,32,33,34,38,39,41] reported obstetric history of women who underwent thyroidectomy for thyroid cancer and revealed that 63 (2.8%) of 2253 women with DTC had at least one miscarriage in their lifetime. In 17 studies [21,22,26,28,29,30,31,32,33,34,35,36,37,38,39,40,41] involving 2337 cases of pregnancy after thyroid cancer treatment, 197 miscarriages (8.2%) were reported. The event rate for miscarriage among cases of pregnancy in the random-effects model was 0.07 (95% CI, 0.05–0.11; I^2^ = 84.1%) (Figure 2A). In three case-control studies [25,30,31], the OR for miscarriage or abortion was 1.80 (95% CI, 1.28–2.53; I^2^ = 33%) in patients with DTC compared with controls (Figure 2B); however, significant publication bias was detected (Egger’s test: *p* = 0.05).

### 3.4. Thyroid Cancer Treatment and Preterm Labor

Five studies [14,25,32,34,38] reported the obstetric history of women who underwent thyroidectomy for thyroid cancer treatment and showed that 134 (3.9%) of 3466 women with DTC experienced preterm labor at least once in their lifetime. In 14 studies [20,21,26,27,30,31,32,34,35,36,37,38,40,42] with 10,237 cases of pregnancy after thyroid cancer treatment, 1167 instances of preterm labor (11.4%) were reported.

**Figure 3:**
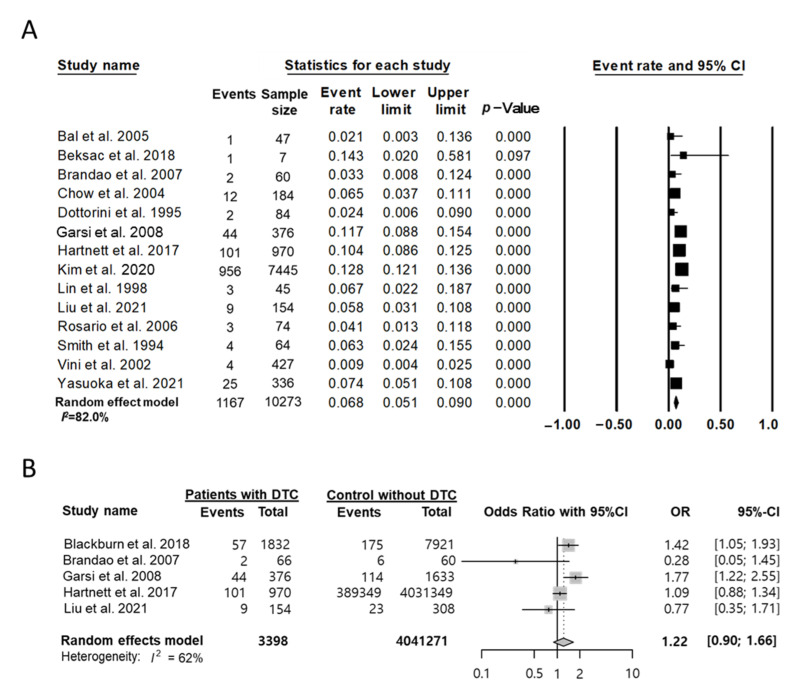
Effect of treatment of DTC on preterm labor. (**A**) Event rate among cases of pregnancy and (**B**) the OR between patients with DTC and controls without DTC. Studies referenced: [20,21,25,26,27,30,31,32,34,35,36,37,38,40,42].

The event rate for preterm labor among cases of pregnancy in the random-effects model was 0.07 (95% CI, 0.05–0.09; I^2^ = 82.0%) (Figure 3A). Five case-control studies [25,27,30,31,42] were included to compare the risk of preterm labor associated with thyroid cancer treatment. The OR for preterm labor was 1.22 (95% CI, 0.90–1.66; I^2^ = 62%) in patients with thyroid cancer when compared with those without thyroid cancer (Figure 3B), which was not significantly different. Publication bias was not detected (Egger’s test: *p* = 0.56).

### 3.5. Thyroid Cancer Treatment and Congenital Anomalies

**Figure 4:**
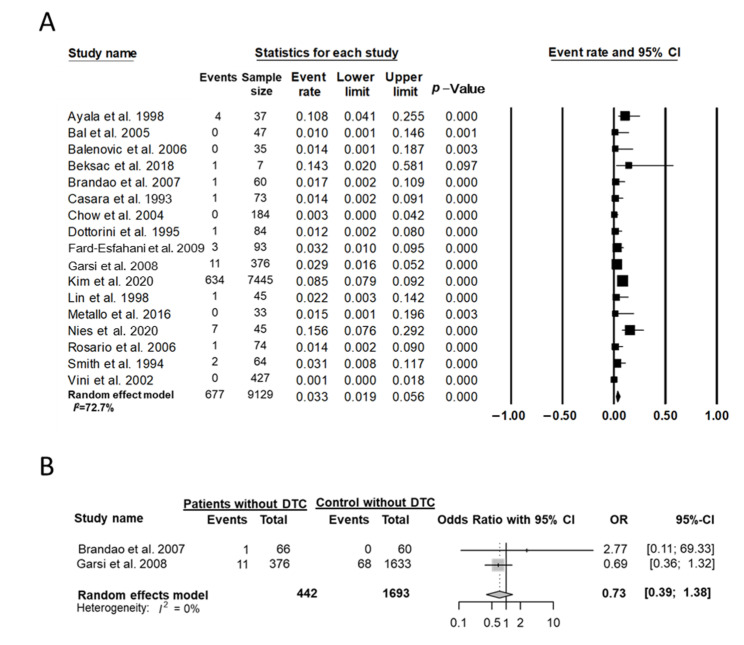
Effect of treatment of DTC on congenital anomalies. (**A**) Event rate among cases of pregnancy and (**B**) the OR between patients with DTC and controls without DTC. Studies referenced: [20,22,26,28,29,30,31,32,33,34,35,36,37,38,39,40,41].

In 17 studies [20,22,26,28,29,30,31,32,33,34,35,36,37,38,39,40,41] with 9129 cases of pregnancy after thyroid cancer treatment, 677 cases of congenital anomalies (7.4%) were reported. The event rate in the random-effects model was 0.03 (95% CI, 0.02–0.06; I^2^ = 72.7%) (Figure 4A). Two case-control studies [30,31] were included to compare the risk of congenital anomalies associated with thyroid cancer treatment (Figure 4B). The OR for the presence of congenital anomalies was 0.73 (95% CI, 0.39–1.38; I^2^ = 0%), which was not significantly different.

### 3.6. Effect of RAIT on Adverse Pregnancy Outcomes

**Figure 5:**
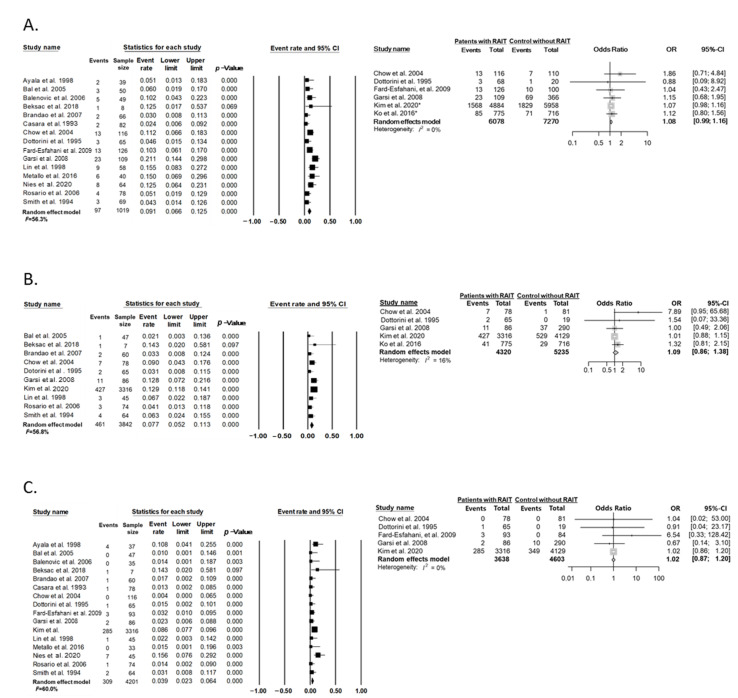
Effect of RAIT on adverse pregnancy outcomes. (**A**) Miscarriage or abortion, (**B**) preterm labor, and (**C**) congenital anomalies. * The study was classified as a case series design because one arm data of patients with thyroid cancer was used in the study. Studies referenced: [14,20,22,26,28,29,30,31,32,33,34,35,36,38,39,40,41].

In 15 studies [22,26,28,29,30,31,32,33,34,35,36,38,39,40,41] involving 1019 cases of pregnancy after RAIT, 97 miscarriages (9.5%) were reported. The event rate among cases of pregnancy in the random-effects model was 0.09 (95% CI, 0.07–0.13; I^2^ = 56.3%) (Figure 5A). The OR for miscarriage or abortion was 1.08 (95% CI, 0.99–1.16; I^2^ = 0%) in patients who received RAIT, as compared with those without RAIT (Figure 5A), which was not significantly different [14,20,29,30,35,36]. Publication bias was not detected (Egger’s test: *p* = 0.29).

In 10 studies [20,26,30,31,32,34,35,36,38,40] with 3842 cases of pregnancy after thyroid cancer treatment, 461 instances of preterm labor (12.0%) were reported. The event rate among cases of pregnancy in the random-effects model was 0.08 (95% CI, 0.05–0.11; I^2^ = 56.8%) (Figure 5B). The OR for preterm labor was 1.09 (95% CI, 0.86–1.38; I^2^ = 16%) in patients who received RAIT when compared with those without RAIT (Figure 5B), which was not significantly different [14,20,30,35,36]. Publication bias was not detected (Egger’s test: *p* = 0.16).

In 16 studies [20,22,26,28,29,30,31,32,33,34,35,36,38,39,40,41] with 4201 cases of pregnancy after thyroid cancer treatment, 309 cases of congenital anomalies (7.3%) were reported. The event rate among cases of pregnancy in the random-effects model was 0.04 (95% CI, 0.02–0.06; I^2^ = 60.0%) (Figure 5C). The risk of the presence of congenital anomalies was not increased in patients with thyroid cancer who received RAIT, as compared with patients who did not receive RAIT (OR, 1.02; 95% CI, 0.87–1.20; I^2^ = 0%) (Figure 5C) [20,29,30,35,36]. Publication bias was not detected (Egger’s test: *p* = 0.69).

**Figure 6:**
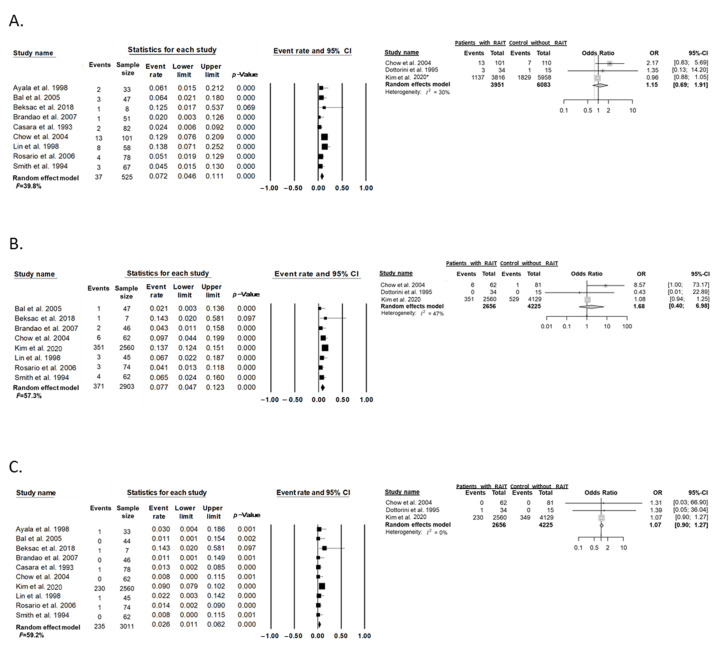
The risk of adverse pregnancy outcomes in patients with an interval of 1 year or more between conception and RAIT. Effect of RAIT on adverse pregnancy outcomes. (**A**) Miscarriage or abortion, (**B**) preterm labor, and (**C**) congenital anomalies. * The study was classified as a case series design because one arm data of patients with thyroid cancer was used in the study. Studies referenced: [14,20,22,26,28,29,30,31,32,33,34,35,36,38,39,40,41].

The subgroup analysis of studies that included patients with an interval of 1 year or more between conception and RAIT revealed that the risk of miscarriage or abortion, preterm labor, and congenital anomalies did not differ between patients who were treated with RAIT and those who were not (Figure 6).

## 4. Discussion

In this meta-analysis, the risk of adverse pregnancy outcomes, including miscarriage, preterm delivery, and congenital anomalies, did not differ between pregnant women with or without thyroid cancer. In the subgroup analysis, RAIT did not increase the risk of adverse pregnancy outcomes in patients with DTC treated with RAIT when compared with those who did not receive RAIT.

In the treatment of DTC, thyroidectomy and RAIT are applied as standard treatments [5]. Total thyroidectomy can lead to postoperative hypothyroidism. In addition, postoperative hypothyroidism reportedly occurs in approximately 30% of patients even after subtotal thyroidectomy [43]. Considering that many patients with DTC undergo thyroid hormone suppression therapy, these patients may experience various thyroid functional statuses (euthyroid, subclinical/overt, hyperthyroid, or hypothyroid) according to the individual thyroid-stimulating hormone target or compliance with levothyroxine [44]. 

Based on studies emphasizing that subclinical hyperthyroidism is not associated with maternal or neonatal complications, the American Thyroid Association recommends that patients with thyroid cancer maintain the same thyroid-stimulating hormone goal before and during pregnancy [5]. Nevertheless, epidemiological studies on the effects of thyroid dysfunction caused by thyroid hormone suppression therapy or thyroidectomy in terms of adverse pregnancy outcomes are lacking [45]. 

This meta-analysis with case-control studies demonstrated an increased risk of miscarriage or abortion, and this result may be biased because of the small number of studies and considering the potentially significant publication bias. In addition, although this meta-analysis showed that thyroid cancer treatment did not increase the risk of preterm labor, significant heterogeneity was noted among the included studies. Two studies showed a higher risk of miscarriage and preterm labor in patients with DTC [25,30]. Blackburn et al. reported a higher incidence of miscarriage and preterm labor in patients with DTC. 

However, the hazard ratio was not significant after adjusting for comorbidities [25]. Garsi et al. also reported that patients with DTC had a significantly higher risk of miscarriage and preterm labor after receiving treatment for DTC than before treatment [30]. Considering the advanced age after treatment compared with that before treatment, the higher incidence of adverse pregnancy outcomes after DTC treatment may be the effect of advanced maternal age [30]. This meta-analysis provides data on the event rates in patients with DTC. 

The event rate for miscarriage was 0.07 in patients with DTC, which is similar to those in the general population from national representative data (0.01–0.18) [46,47,48]. Four European case series studies [22,33,39,41] and one Indian case series study [34], which reported the obstetric history of women with DTC, showed that the prevalence of miscarriage at least once in their lifetime was similar to that in the general population in the EPIC study [49] or general Indian population [50]. The events rates for preterm labor were 0.07 in patients with DTC, which were similar to those in the general population (0.06–0.23) [51,52,53,54].

The event rates for congenital anomalies were 0.03 in patients with DTC, which were similar to those in the general population (0.01–0.03) [51,52,55,56]. Nonetheless, a large population-based study conducted by Kim et al. revealed a higher risk of congenital anomalies in women with DTC compared with that in the general population from the Korean National Health Insurance Service. The study by Kim et al. included more pregnant women aged >35 years than the study on the general population (34% vs. 15.9%), which could have resulted in a higher risk [20,56]. Although we could not perform subgroup analysis according to thyroid functional status, the present study provides substantial evidence that thyroid cancer treatment does not increase the risk of adverse pregnancy outcomes compared to women without DTC.

RAIT is known to be able to affect gonadal tissues [16,17,42]. In men, an association between RAIT and a transient reduction in sperm count, elevated follicle-stimulating hormone (FSH) levels, and testicular damage have been reported [16,57]. A recent longitudinal prospective study revealed a statistically significant increase in the number of chromosomal abnormalities in sperm at 3 and 13 months after RAIT with 100 mCi [58]. Therefore, contraception is usually recommended at least for 3 months in men after RAIT [5]. In addition, high radioactive iodine (RAI) activities of 500–800 mCi increased the risk of sustained elevation of FSH [5]. 

Therefore, the American Thyroid Association (ATA) recommends sperm banking for men who need cumulative RAI activities greater than 400 mCi [5,59]. Proper hydration, frequent urination, and avoidance of constipation may also be helpful in reducing radiation exposure to the gonads [60]. In women, RAIT has been reported to be associated with oligomenorrhea, transient secondary amenorrhea, and premature menopause [5]. 

About 12–31% of menstrual irregularities and 8–16% of amenorrhea [17] or a significant decrement of anti-Müllerian hormone (AMH) [18] in the first year after RAIT have been reported. Although, many previous epidemiologic studies have not found conclusive evidence for decreased fertility in these women [18,30,34,35,61], there is significant heterogeneity between studies. Research reported that RAIT was associated with delayed childbearing and reduced birthrates in a specific population of advanced age (>35 years) [19]. Therefore, in women over 35 years of age with low-risk DTC, RAIT should be carefully considered when planning pregnancy [19,62]. 

These women should be informed and counseled about the potential deleterious effects on fertility and fertility [63]. AMH measurement is suggested as a good option to estimate ovarian reserve for fertility patients in RAIT decision-making process, although it cannot fully estimate the risk of infertility [62]. As suggested by the American Society of Clinical Oncology, interventions for preserving fertility, including oocyte cryopreservation, may be useful particularly in women with a limited ovarian reserve [63,64], although its evidence in women with RAIT remains lacking. Further studies are warranted.

The ATA recommends that reproductive-age women receiving RAIT should undergo negative screening evaluation for pregnancy and should avoid pregnancy for 6–12 months after receiving RAI [5]. Despite these recommendations, RAI may inadvertently be administered to pregnant women because of a clinician’s negligence or false-negative pregnancy test results [65]. The effects of inadvertent exposure on embryos and fetuses vary depending on the pregnancy stage and absorbed RAI dose [65]. Exposure to RAI during the very early stage of pregnancy may result in cellular damage and embryo death although it is unlikely to induce congenital anomalies in the surviving embryos [66]. 

At 3–7 weeks after conception, exposure to RAI can lead to congenital anomalies, such as microcephaly, cleft palate, and genital deformities [66]. Considering that the thyroid gland is formed by 10–12 weeks of gestation, exposure to RAI after 10 weeks of gestation can result in fetal thyroid ablation [67,68,69]. Exposure after 8 weeks of gestation can impair the central nervous system. In particular, mental retardation has been frequently reported with exposure at 8–25 weeks after conception [70,71]. Additionally, exposure to RAI can increase the risk of some cancers, such as leukemia, skin cancer, lung cancer, breast cancer, and thyroid cancer [71,72]. 

When inadvertent exposure occurs, potassium iodide can be helpful in reducing fetal exposure to RAI within 12 h of RAI administration [65,73]. However, data on therapeutic abortion are limited [74]. During pregnancy, congenital anomalies should be closely monitored. Levothyroxine supplementation should be considered to maintain maternal thyroid hormone levels at the high end of the normal range. For neonates, thyroid function should be evaluated, and levothyroxine supplementation should be initiated to prevent any neurological impairment [65].

This meta-analysis provides data on the event rates in patients who received RAIT. The event rates in patients receiving RAIT were 0.09, 0.08, and 0.04 for miscarriage, preterm labor, and congenital anomalies, respectively, which were similar to those observed in the general population [46,47,48,51,52,53,54,55,56]. In addition, this meta-analysis with case-control studies showed that RAIT did not increase the risk of miscarriage, preterm labor, and congenital anomalies without significant heterogeneity among the included studies compared with those with DTC who did not receive RAIT.

The strengths of this study include the collection of evidence through a rigorous systematic review and meta-analysis. However, the present study has certain limitations. We could not adjust for the complications of DTC treatment, including hyperparathyroidism, the stage of DTC, and recurrence, because corresponding data were unavailable. In addition, a subgroup analysis according to total thyroidectomy or hemilobectomy, thyroid functional status, and RAIT dosage was not conducted due to the lack of data.

## 5. Conclusions

The meta-analysis results suggest that thyroid cancer treatment is not associated with an increased risk of adverse pregnancy outcomes. In particular, RAIT after thyroidectomy was not found to increase the risk of adverse pregnancy outcomes in patients with DTC compared with those with DTC who did not receive RAIT.

## Figures and Tables

**Table 1:** Study characteristics of the included studies.

Study [Reference]	Region	Study Design	Participants	No. of Total Participants	No. of Patients with RAI	Age	Pregnant Outcomes
Liu et al. 2021 [42]	China	Case-control design	Data form the University Hospital in Beijing	562 women Cases: 154 women with thyroid cancer Controls: 308 matched controls without thyroid cancer	5 women	Median age at pregnancy: Patients with thyroid cancer: 33 Controls: 32	Pregnant women with thyroid cancer compared to those without thyroid cancer Preterm delivery: 5.84% vs. 7.47% Adjusted OR with 95% CI (Reference group: women without thyroid cancer) Preterm delivery: 0.73 (0.32–1.67)
Yasuoka et al. 2021 [21]	Japan	Case series design	Data from major tertiary institutions in Japan	341 women with thyroid cancer	NA	NA	Miscarriage: 5 of 314 pregnancies Preterm delivery: 25 of 314 pregnancies
Kim et al. 2020 [20]	Korea	Case series design *	Data from Health Insurance Review and Assessment database	10,842 pregnancies in women with thyroid cancer Cases :4884 with RAITControls: 5958 without RAIT	4884 pregnancies	Mean (SD): 33.3 (4.4)	Pregnant women without RAI compared to those with RAI Abortion: 30.7% vs. 32.1% Preterm deliveries: 12.8% vs. 12.9% Congenital malformations: 8.9% vs. 9.0% Adjusted OR with 95% CI (Reference group: patients with RAI dose ≤1.11 GBq) Abortion 1.12–3.7 GBq: 1.11 (0.91–1.36) ≥3.8 GBq: 1.02 (0.85–1.22) Preterm delivery 1.12–3.7 GBq: 0.79 (0.58–1.08) ≥3.8 GBq: 0.82 (0.63–1.08) Congenital malformation 1.12–3.7 GBq: 1.06 (0.72–1.56) ≥3.8 GBq: 1.08 (0.76–1.52)
Nies et al. 2020 [22]	The Netherlands	Case series design	A nationwide, long-term follow-up study on childhood differentiated thyroid cancer in the Netherlands	56 women with thyroid cancer (64 pregnancies)	56 women	Median age at first pregnancy (IQR) 25.5 (22.5–30.0)	Miscarriage: 8 of 56 women (64 pregnancies) after RAIT Congenital malformation: 7 of 45 pregnancies after RAIT
Blackburn et al. 2018 [25]	USA	Case-control design	The Utah Population Database, which links data from the Utah Cancer Registry	9753 women Cases: 1832 women with thyroid cancer Controls 7921 matched control without thyroid cancer)	947 women	Median age 36	Miscarriage: 25 of 1832 women by 1–5 years after thyroid cancer diagnosis and 63 of 7921 women in general population cohort Preterm deliveries: 57 of 1832 women by 1–5 years after thyroid cancer diagnosis and 175 of 7921 women in general population cohort
Beksaç et al. 2018 [26]	Turkey	Case series design	The clinical records of 8 pregnant women who received treatment for PTC before their pregnancy	8 women with thyroid cancer	8 women	Mean age 34.3 years	Miscarriage: 1 of 8 pregnancies after thyroid cancer treatment Preterm delivery: 1 or 7 pregnancies after thyroid cancer treatment Congenital malformation: 1 or 7 pregnancies after thyroid cancer treatment
Hartnett et al. 2017 [27]	USA	Case-control design	Cancer registries in the states of Georgia, North Carolina, and Tennessee	4,032,219 women Cases: 970 women with thyroid cancer Controls: 4,031,349 matched control without cancer	NA	NA	Adjusted risk ratio (95% CI) for preterm deliveries: 1.0 (0.8, 1.2)
Metallo et al. 2016 [28]	France	Case series design *	Data form the University Hospital in Nancy	45 women with thyroid cancer RAIT ≤3.85 GBq: 18 women >3.85 GBq: 27 women	45 women	Mean (SD) Patients with RAIT ≤3.85 GBq: 27.7 (6.7) >3.85 GBq: 36.1 (11.1)	Miscarriage: 1of 18 patients with RAIT ≤3.85 GBq and 5 of 27 patients with RAIT >3.85 GBq Congenital anomaly was not observed in this study.
Ko et al. 2016 [14]	Taiwan	Case series design *	The National Health Insurance Research Database in Taiwan	1491 women with thyroid cancer Cases: 775 patients with RAIT Controls: 716 without RAIT	775 women	NA	Abortion: 71 of 716 patients with RAIT and 85 of 775 patients without RAIT Preterm delivery: 29 of 716 patients with RAIT and 41 of 775 patients without RAIT Adjusted HR (95% CI) (patients without RAIT vs. patients with RAIT) Abortion: 0.67 (0.49–0.93) Preterm delivery: 1.32 (0.81–2.15)
Fard-Esfahani et al. 2009 [29]	Iran	Case series design *	Data from one institution in Iran	227 pregnancies in women with thyroid cancer Cases: 126 pregnancies after RAIT Controls: 101 pregnancies before RAIT	126 pregnancies in 100 women	NA	Miscarriage: 13 of 126 pregnancies after I^131^ treatment (10/100 women) and 17 of 101 pregnancy before thyroid cancer treatment
Garsi et al. 2008 [30]	France and Italy	Case-control design	Data from three institutions in France and one institution in Italy	2673 pregnancies in 1126 patients with thyroid cancer Cases: 595 pregnancies after thyroid cancer treatment Controls: 2078 pregnancies before thyroid cancer treatment,	483 pregnancies	NA	Miscarriage: 193 of 1854 pregnancies before thyroid cancer treatment, 92 of 475 pregnancies after thyroid cancer treatment (75 of 389 pregnancies after I^131^ treatment) Preterm delivery: 114 of 1633 live births before thyroid cancer treatment, 44 of 376 live births after thyroid cancer treatment (40 of 309 live births after I^131^ treatment) Congenital malformation: 68 of 1633 live births before thyroid cancer treatment, 11 of 376 live births after thyroid cancer treatment (9 of 309 live births after I^131^ treatment)
Brandao et al. 2007 [31]	Brazil	Case-control design	Data from three institutions in Brazil	126 pregnancies Cases: 66 pregnancies after RAIT Controls: 60 pregnancies in healthy women	66 pregnancies (48 women)	NA	Abortion: 6/66 pregnancies after RAIT and 7/60 pregnancies in healthy women Preterm delivery: 1/66 pregnancies after RAIT and 6/60 pregnancies in healthy women Congenital malformation: 1/66 pregnancies after RAIT. Congenital malformation was not observed in healthy women
Rosário et al. 2006 [32]	Brazil	Case series design	Data from one institution in Brazil	78 pregnancies after RAIT	78 pregnancies	NA	Miscarriage: 4 of 78 pregnancies after RAIT Preterm delivery: 3 or 78 pregnancies after RAIT Congenital malformation: 1 or 78 pregnancies after RAIT
Balenovic et al. 2006 [33]	Croatia	Case series design	Data from one institution in Croatia	26 women after RAIT (40 pregnancies)	26 women (40 pregnancies)	NA	Miscarriage: 2 of 26 women after RAIT (5 of 40 pregnancies) Congenital malformation was not observed (0/35 births)
Bal et al. 2005 [34]	India	Case series design	Data from one institution in India	50 pregnancies after RAIT in 40 women	50 pregnancies	NA	Miscarriage: 3 of 50 pregnancies after RAIT Preterm delivery: 1 or 50 pregnancies after RAIT Congenital malformation was not observed
Chow et al. 2004 [35]	China	Case series design *	Data from one institution in China	263 pregnancies in 104 women after thyroid cancer treatment Cases: 143 pregnancies after RAIT Controls: 110 pregnancies without RAIT	143 pregnancies	Mean age (SD) at pregnancy: No RAI: 26.5 (5.4) RAI scanning dose: 30.7 (4.7)RAI ablation does: 31.4 (4.6)	Miscarriage: 18 of 143 pregnancies with RAIT (13 of 116 pregnancies with I^131^ ablative dose) and 7 of 110 pregnancies without RAIT Preterm delivery: 11 of 143 pregnancies with RAIT (7 of 116 pregnancies with I^131^ ablative dose) and 1 of 110 pregnancies without RAITCongenital malformation was not observed
Vini et al. 2002 [37]	UK	Case series design	Data from one institution in UK	441 pregnancies after thyroid cancer treatment (276 women)	441 pregnancies (276 women)	NA	Miscarriage:14 of 441 pregnancies after RAIT Preterm delivery: 4 of 427 pregnancies after RAIT Congenital malformation: 0 of 427 pregnancies after RAIT
Lin et al. 1998 [38]	Taiwan	Case series design	Data from one institution in Taiwan	58 pregnancies after I^131^ treatment (37 women)	58 pregnancies	Mean age at pregnancy (SD): 27.97 (3.49)	Miscarriage: 8 of 58 pregnancies after RAIT Preterm delivery: 3 of 58 pregnancies after RAIT Congenital malformation: 1 of 58 pregnancies after RAIT
Ayala et al. 1998 [39]	Spain	Case series design	Data from one institution in Spain	39 pregnancies after I^131^ treatment (26 women)	39 pregnancies	Mean age at the time of the first pregnancy: 26.9	Miscarriage: 2 of 39 pregnancies after RAIT Congenital malformation: 4 of 39 pregnancies after RAIT
Dottorini et al. 1995 [36]	Italy	Case series design *	Data from one institution in Italy	84 pregnancies in 64 women with thyroid cancer Cases: 65 pregnancies after RAIT Controls: 19 pregnancies without RAIT	65 pregnancies	NA	Miscarriage: 3 of 65 pregnancies with RAIT and 1 of 19 pregnancies without RAIT Preterm delivery: 2 of 65 pregnancies with RAIT and 0 of 19 pregnancies without RAIT Congenital malformation: 1 of 65 pregnancies with RAIT and 0 of 19 pregnancies without RAIT
Smith et al. 1994 [40]	USA	Case series design	Review of The University of Texas M. D. Anderson Cancer Center Tumor Registry	69 pregnancies in 32 women after RAIT	69 pregnancies	Mean age at I^131^ treatment: 18.3	Miscarriage: 3 of 69 pregnancies after RAIT Preterm delivery: 4 of 69 pregnancies after RAIT Congenital malformation: 2 of 69 pregnancies after RAIT
Casara et al. 1993 [41]	Italy	Case series design	Data from one institution in Italy	70 women with RAIT	70 women	Mean age (SD) at pregnancy: 29 (4.2)	Miscarriage: 2 of 75 pregnancies after RAIT Congenital malformation: 1 of 73 live births

* The study was classified as a case series design because one arm data of patients with thyroid cancer was used in the study.

## Data Availability

The data presented in this study are available upon reasonable request from the corresponding author.

## Supplementary Materials

The following supporting information can be downloaded at: https://www.mdpi.com/article/10.3390/cancers14102382/s1, Table S1. PRISMA checklist; Table S2. Quality of the included studies according to the Newcastle–Ottawa Quality Assessment Scale.
